# Comparison of essential medicines lists in 137 countries

**DOI:** 10.2471/BLT.18.222448

**Published:** 2019-04-04

**Authors:** Nav Persaud, Maggie Jiang, Roha Shaikh, Anjli Bali, Efosa Oronsaye, Hannah Woods, Gregory Drozdzal, Yathavan Rajakulasingam, Darshanand Maraj, Sapna Wadhawan, Norman Umali, Ri Wang, Marcy McCall, Jeffrey K Aronson, Annette Plüddemann, Lorenzo Moja, Nicola Magrini, Carl Heneghan

**Affiliations:** a Centre for Urban Health Solution, St. Michael’s Hospital, 80 Bond Street, Toronto, Ontario, M5B 1X2, Canada.; b Centre for Evidence-Based Medicine, University of Oxford, Oxford, England.; c Department of Essential Medicines and Health Products, World Health Organization, Geneva, Switzerland.

## Abstract

**Objective:**

To compare the medicines included in national essential medicines lists with the World Health Organization’s (WHO’s) *Model list of essential medicines*, and assess the extent to which countries’ characteristics, such as WHO region, size and health care expenditure, account for the differences.

**Methods:**

We searched the WHO’s Essential Medicines and Health Products Information Portal for national essential medicines lists. We compared each national list of essential medicines with both the 2017 WHO model list and other national lists. We used linear regression to determine whether differences were dependent on WHO Region, population size, life expectancy, infant mortality, gross domestic product and health-care expenditure.

**Findings:**

We identified 137 national lists of essential medicines that collectively included 2068 unique medicines. Each national list contained between 44 and 983 medicines (median 310: interquartile range, IQR: 269 to 422). The number of differences between each country’s essential medicines list and WHO’s model list ranged from 93 to 815 (median: 296; IQR: 265 to 381). Linear regression showed that only WHO region and health-care expenditure were significantly associated with the number of differences (adjusted *R^2^*: 0.33; *P* < 0.05). Most medicines (1248; 60%) were listed by no more than 10% (14) of countries.

**Conclusion:**

The substantial differences between national lists of essential medicines are only partly explained by differences in country characteristics and thus may not be related to different priority needs. This information helps to identify opportunities to improve essential medicines lists.

## Résumé

**Objectif:**

Comparer les médicaments inclus dans les listes nationales des médicaments essentiels avec la Liste modèle de l'Organisation mondiale de la Santé (OMS) des médicaments essentiels, et déterminer dans quelle mesure les caractéristiques des pays, comme la région OMS, la taille et les dépenses de soins de santé, expliquent les différences.

**Méthodes:**

Nous avons recherché des listes nationales des médicaments essentiels sur le Portail d'information – Médicaments essentiels et produits de santé de l'OMS. Nous avons comparé chaque liste nationale des médicaments essentiels avec la Liste modèle de l'OMS de 2017 et d'autres listes nationales. Nous avons utilisé une régression linéaire pour déterminer si les différences dépendaient de la région OMS, de la taille de la population, de l'espérance de vie, de la mortalité infantile, du produit intérieur brut et des dépenses de soins de santé.

**Résultats:**

Nous avons sélectionné 137 listes nationales des médicaments essentiels qui contenaient au total 2068 médicaments différents. Chaque liste national recensait entre 44 et 983 médicaments (médiane: 310; intervalle interquartile, IQR: de 269 à 422). Le nombre des différences entre la liste des médicaments essentiels de chaque pays et la Liste modèle de l'OMS variait entre 93 et 815 (médiane: 296; IQR: de 265 à 381). La régression linéaire a montré que seules la région OMS et les dépenses de soins de santé étaient clairement associées au nombre des différences (*R^2^* ajusté: 0,33; *P* < 0,05). La plupart des médicaments (1248; 60%) étaient listés par 10% (14) des pays, pas plus.

**Conclusion:**

Les différences importantes entre les listes nationales des médicaments essentiels s'expliquent seulement en partie par des différences au niveau des caractéristiques des pays et ne sont donc pas forcément liées à différents besoins prioritaires. Ces informations aident à identifier des possibilités d'améliorations des listes des médicaments essentiels.

## Resumen

**Objetivo:**

Comparar los medicamentos incluidos en las listas nacionales de medicamentos esenciales con la Lista Modelo de Medicamentos Esenciales de la Organización Mundial de la Salud (OMS) y evaluar en qué medida las diferencias se deben a las características de los países, como la región, el tamaño y el gasto en atención sanitaria de la OMS.

**Métodos:**

Se realizaron búsquedas en el Portal de Información de Medicamentos Esenciales y Productos de Salud de la OMS para obtener listas nacionales de medicamentos esenciales. Se comparó cada lista nacional de medicamentos esenciales con la Lista Modelo de la OMS de 2017 y con otras listas nacionales. Se utilizó la regresión lineal para determinar si las diferencias dependían de la región de la OMS, el tamaño de la población, la esperanza de vida, la mortalidad infantil, el producto interno bruto y el gasto en atención sanitaria.

**Resultados:**

Identificamos 137 listas nacionales de medicamentos esenciales que incluían colectivamente 2068 medicamentos únicos. Cada lista nacional contenía entre 44 y 983 medicamentos (mediana 310: rango intercuartil, IQR: 269 a 422). El número de diferencias entre la lista de medicamentos esenciales de cada país y la Lista Modelo de la OMS osciló entre 93 y 815 (mediana: 296; IQR: 265 a 381). La regresión lineal mostró que sólo la región de la OMS y el gasto en atención sanitaria se asociaron significativamente con el número de diferencias (R2 ajustado: 0,33; P < 0,05). La mayoría de los medicamentos (1248; 60 %) fueron listados por no más del 10 % (14) de los países.

**Conclusión:**

Las diferencias sustanciales entre las listas nacionales de medicamentos esenciales sólo se explican en parte por las diferencias en las características de los países y, por lo tanto, pueden no estar relacionadas con las diferentes necesidades prioritarias. Esta información ayuda a identificar oportunidades para mejorar las listas de medicamentos esenciales.

## ملخص

**الغرض:**

مقارنة الأدوية المدرجة في قوائم الأدوية الأساسية الوطنية، مع القائمة النموذجية للأدوية الأساسية التابعة لمنظمة الصحة العالمية (WHO)، وتقييم مدى تفسير خصائص هذه البلدان للاختلافات المكتشفة، وتشمل هذه الخصائص منطقة منظمة الصحة العالمية، وحجم هذه البلدان ونفقات الرعاية الصحية بها،.

**الطريقة:**

قمنا بالبحث في بوابة معلومات الأدوية الأساسية والمنتجات الصحية لمنظمة الصحة العالمية للتعرف على قوائم الأدوية الأساسية الوطنية. وقمنا بمقارنة كل قائمة وطنية للأدوية مع كل من القائمة النموذجية لمنظمة الصحة العالمية عام 2017، وكذلك مقارنتها بقوائم الأدوية الوطنية الأخرى. واستخدمنا التحوف الخطي لتحديد ما إذا كانت الاختلافات تعتمد على منطقة منظمة الصحة العالمية، وتعداد السكان، والعمر المتوقع، ووفيات الرضع، والناتج المحلي الإجمالي، ونفقات الرعاية الصحية.

**النتائج:**

حددنا 137 قائمة وطنية من الأدوية الأساسية التي تضم مجتمعة 2068 من الأدوية الفريدة. احتوت كل قائمة وطنية على ما بين 44 و983 دواءً (المتوسط ​​310: المدى الربيعي، IQR: 269 إلى 422). تراوح عدد الاختلافات بين قائمة الأدوية الأساسية لكل بلد والقائمة النموذجية لمنظمة الصحة العالمية، بين 93 و815 (المتوسط: 296؛ المدى الربيعي: 265 إلى 381). أظهر التحوف الخطي أن كل من منطقة منظمة الصحة العالمية والإنفاق على الرعاية الصحية، وحدهما فقط مرتبطين بشكل كبير بعدد الاختلافات (نسبة *R ^2 ^* المعدلة: 0.33؛ *نسبة الاحتمال * > 0.05). تم إدراج معظم الأدوية (1248؛ نسبة 60%) بواسطة ما لا يزيد عن 10% (14) من البلدان.

**الاستنتاج:**

لا يمكن تفسير الاختلافات الجوهرية بين القوائم الوطنية للأدوية الأساسية على أساس الاختلافات في خصائص البلدان إلا بشكل جزئي، وبالتالي فقد لا تكون مرتبطة الاحتياجات ذات الأولوية المختلفة. وتساعد هذه المعلومات على تحديد فرص تحسين قوائم الأدوية الأساسية. ****

## 摘要

**目的:**

将国家基本药物清单包含药物与《世界卫生组织基本药物标准清单》进行比较，并评估各国特征（例如世卫组织区域、规模和医疗保健支出）间的差异程度。

**方法:**

我们在世界卫生组织的基本药物和卫生产品信息门户网站对国家基本药物清单进行了搜索。我们将各国基本药物清单与 2017 年世卫组织标准清单以及其他国家清单均进行了比较。我们采用线性回归的分析方法来确定差异是否取决于世卫组织区域、人口规模、预期寿命、婴儿死亡率、国内生产总值和医疗保健支出等因素。

**结果:**

我们确定了 137 国的基本药物清单，这些清单共计包括 2068 种独特药物。各国清单包含 44 至 983 种药物（中值 310：四分位距，IQR：269 至 422）。各国基本药物清单与世卫组织标准清单间的差异数值范围为 93 至 815（中值：296；IQR：265 至 381）。线性回归显示，仅世卫组织区域和医疗保健支出与差异数值显著相关（调整后的 *R^2^*：0.33；P < 0.05）。仅有不超过 10% 的国家（14 个）将大多数药物（1248 种；占 60％）列入清单。

**结论:**

国家特征差异只是国家基本药物清单之间实质性差异的部分原因，因此，这可能与不同的优先需求无关。此类信息有助于发现改进基本药物清单的机会。****

## Резюме

**Цель:**

Сравнить лекарственные препараты, включенные в национальные перечни основных лекарственных средств с Примерным перечнем Всемирной организации здравоохранения (ВОЗ) основных лекарственных средств, и оценить, в какой мере характеристики страны, такие как регион ВОЗ, размеры и уровень затрат на здравоохранение, влияют на различия между ними.

**Методы:**

Авторы провели поиск на информационном портале ВОЗ, посвященном основным лекарственным средствам и медицинской продукции, с целью найти там национальные перечни основных лекарственных средств. Каждый такой национальный перечень основных лекарственных средств сравнивался с Примерным перечнем ВОЗ 2017 года и с перечнями других стран. Авторы использовали линейную регрессию для определения того, зависели ли различия от региона ВОЗ, размера популяции, ожидаемой продолжительности жизни, детской смертности, валового национального продукта и затрат на здравоохранение.

**Результаты:**

Было найдено 137 национальных перечней основных лекарственных средств, которые в совокупности включали 2068 уникальных лекарственных средств. В каждом национальном перечне было от 44 до 983 лекарственных средств (медианное значение 310, межквартильный размах (МКР): от 269 до 422). Количественно различие между перечнем основных лекарственных средств для каждой отдельной страны и Примерным перечнем ВОЗ основных лекарственных средств составляло от 93 до 815 наименований (медианное значение 296, МКР: от 265 до 381). Линейная регрессия показала, что только регион ВОЗ и затраты на здравоохранение были в значительной мере связаны с количеством наблюдаемых различий (скорректированное значение R2: 0,33; P < 0,05). Большая часть лекарственных средств (1248, 60%) входила в перечни не более чем 10% стран (14 стран).

**Вывод:**

Существенные различия между национальными перечнями основных лекарственных средств лишь частично объясняются различными характеристиками государств и, следовательно, могут быть не связаны с разными приоритетными потребностями. Данная информация помогает выявить возможности для улучшения перечней основных лекарственных средств.

## Introduction

More than 5 billion people live in countries that use essential medicines lists. These lists typically contain hundreds of medicines intended to meet the priority health-care needs of a population.^1^^–^^3^ Since the World Health Organization (WHO) published the first *Model list of essential medicines* in 1977, the list has been revised every two years and adapted to circumstances in more than one hundred countries. Governments and health-care institutions use essential medicines lists to determine which medicines to fund, stock, prescribe and dispense.^4^ As essential medicines lists influence the medicines that people have access to, contents of these lists constitute important determinants of health worldwide.

Countries must select medicines for their essential lists appropriately to facilitate sustainable, equitable access to medicines and promote their appropriate use.^4^ Since a country’s list is intended to meet the needs of its population, countries that are geographically close or similar to each other in population size, health-care expenditure and health status might be expected to have similar essential medicines lists. Differences between such lists that are not explained by differences in country-specific needs may represent opportunities for improving the lists.

Here we aimed to compare the medicines included in national essential medicines lists with the 2017 WHO’s *Model list of essential medicines*,^5^ and to determine whether characteristics, such as WHO Region, population size, and health-care expenditure account for the differences.

## Methods

We prespecified the main analysis for this observational study before data collection (NCT03218189) and report the results using the STROBE reporting guidelines.^6^^,^^7^

In June 2017, we searched the WHO essential medicines and health products information portal. This online repository contains hundreds of publications on medicines and health products related to WHO priorities and has a full section dedicated to national lists of essential medicines.^2^^,^^3^ A WHO information specialist actively searched for updated versions of national lists, including national formularies, reimbursement lists and lists based on standard treatment guidelines. We included all national lists of essential medicines that were posted on the repository irrespective of publication date and language. When we found more than one national list from the same country, we used the most recent list.

We excluded documents that were not essential medicines lists, such as prescribing guidelines. We also excluded diagnostic agents, antiseptics, disinfectants and saline solutions.

### Data collection processes

We developed a data extraction method for medicines in national lists, which we pilot-tested on lists from five countries. One of six reviewers extracted information from each country and another reviewer verified the information before inclusion in an electronic database.

For identified countries with essential medicines lists, we collected eight country characteristics that might explain differences in the lists and that are widely available and commonly used in international comparisons: WHO region; population size; life expectancy; infant mortality; gross domestic product (GDP) per capita; health care-expenditure per capita; GINI index as a measure of income inequality; and the corruption perception index. In June 2017, we extracted data on WHO Region and per capita health-care expenditure from the WHO Global Health Observatory, the most recent information available at the time.^8^ We extracted data on population, life expectancy, infant mortality and GDP per capita from the Central Intelligence Agency’s World Factbook.^9^ We obtained the GINI index from the most recent data available from the World Bank in the United Nations Human Development Report 2016.^10^ We retrieved the corruption perception score from Transparency International’s 2016 corruption perceptions index.^11^

### Data extraction

From each country’s list we abstracted medicines using International Nonproprietary Names (INNs).^12^ For medicines whose names were not in English, we used the Anatomical Therapeutic Chemical classification system,^13^ if available, or translated the names using Google Translate.^14^ We listed each medicine individually, whether it was part of a combination product or not. We treated medicine bases and their salts (e.g. promethazine hydrochloride and promethazine) as the same medicines, as well as different compounds of the same vitamin or mineral (e.g. ferrous fumarate and ferrous sulfate).

We used the Anatomical Therapeutic Chemical code for each medicine and the Anatomical Therapeutic Chemical structure to determine the level of relatedness between medicines: level 1, anatomical main group (e.g. metformin is “A” for alimentary tract); level 2, therapeutic subgroup (e.g. metformin is “A10” for alimentary tract medicines used to treat diabetes); level 3, pharmacological subgroup (e.g. metformin is “A10B” for alimentary tract medicines used to treat diabetes that lower blood glucose); level 4, chemical subgroup (e.g. metformin is “A10BA” for alimentary tract medicines used to treat diabetes that lower blood glucose that are biguanides).^15^ WHO’s model list indicates (with a square box) that some listed medicines are merely exemplars of several medicines that should be considered therapeutically equivalent.^5^ We assumed that the medicines in the same chemical subgroup as the exemplar were equivalent (e.g. enalapril is equivalent to all other in the chemical subgroup C09A: captopril, lisinopril, perindopril, ramipril, quinapril, benazepril, cilazapril, fosinopril, trandolapril, spirapril, delapril, moexipril, temocapril, zofenopril and imidapril), except when WHO’s list specified particular equivalent medicines (e.g. bisoprolol is specified as equivalent to atenolol, metoprolol, and carvedilol). As a result of uncertainty whether these medicines are truly equivalent, and because we do not know how countries interpreted the indications of equivalence, or if they used them at all, we also report results disregarding the equivalence to exemplars.

### Data analysis

For descriptive data, we calculated medians with interquartile ranges (IQRs).

#### Comparison with WHO’s model list

To determine whether countries’ characteristics accounted for differences between each country’s list and the 2017 WHO model list, we created a linear regression model with the total number of differences from the WHO’s model list as the dependent variable and the following characteristics as independent variables: WHO region, population size, life expectancy, infant mortality, GDP per capita, and health-care expenditure per capita. We had to exclude the variables inequality and corruption perception, since only 95 (69 %) countries had available information. We present the adjusted *R^2^* values for the number of independent variables. We conducted several post-hoc sensitivity analyses: removed longer lists to assess the effect of outliers, employed the Tanimoto coefficient that accounts for list length and used the 2015 WHO model list instead of the 2017 list as a reference to allow for a delay in updating national lists.^16^ We used R statistical package (R Foundation, Vienna, Austria).

#### Country comparisons

To calculate a similarity score, we divided medicines into those that are commonly listed (by at least 50% of countries) and those that are uncommonly listed (by less than 50% of countries). For each country’s list we calculated the score by counting the medicines on that list that are commonly listed and subtracting the number of uncommonly listed medicines. This calculation provides a similarity integer score for each country; positive scores indicate that most medicines in the country’s list are commonly listed in other countries’ lists, and negative scores indicate that most medicines are uncommonly listed in other countries’ lists.

### Data sharing

The underlying data used in this study are publicly available and, separately, a database with updated information about national essential medicines lists will be maintained online.^17^^,^^18^

## Results

We identified essential medicines lists posted on the WHO repository for 137 countries (70% of 195 countries). The total number of medicines on each country’s list ranged from 44 to 983 (median: 310; IQR: 269 to 422). In total we identified 2068 unique medicines. Table 1 (available at: http://www.who.int/bulletin/volumes/97/6/18-222448) presents the characteristics of the included countries.

**Table 1:** National lists of essential medicines in 137 countries

Country	GDP per capita in 2017, Intl $	Health expenditure per capita in 2014, Intl $	Year of list	Total no. of medicines on list	Similarity with WHO Model List, no. (%)^a^	Dissimilarity with WHO Model List, no.^b^	Similarity score
**Afghanistan**	2 000	167	2014	258	196 (78)	62	104
**Albania**	12 500	615	2011	214	121 (57)	93	26
**Algeria**	15 200	932	2016	445	161 (36)	284	−145
**Angola**	6 800	239	2008	64	51 (80)	13	40
**Antigua and Barbuda**	26 400	1208	2007	292	208 (71)	84	140
**Argentina**	20 900	1137	2011	468	285 (61)	183	0
**Armenia**	9 500	362	2010	267	234 (88)	33	129
**Bahrain**	49 000	2273	2015	550	271 (49)	279	−106
**Bangladesh**	4 200	88	2008	187	170 (91)	17	129
**Barbados**	18 600	1014	2011	625	266 (43)	359	−159
**Belarus**	18 900	1031	2012	371	192 (52)	179	−53
**Belize**	8 300	489	2008	370	253 (68)	117	78
**Bhutan**	9 000	281	2016	291	202 (69)	89	89
**Bolivia (Plurinational State of)**	7 600	427	2011	352	248 (70)	104	92
**Bosnia and Herzegovina**	12 800	957	2009	181	107 (59)	74	29
**Botswana**	17 000	871	2012	340	233 (69)	107	108
**Brazil**	15 600	1318	2014	405	235 (58)	170	−49
**Bulgaria**	21 800	1399	2011	361	114 (32)	247	−171
**Burkina Faso**	1 900	82	2014	274	217 (79)	57	124
**Burundi**	700	58	2012	293	204 (70)	89	97
**Cabo Verde**	7 000	310	2009	564	295 (52)	269	−78
**Cambodia**	4 000	183	2003	44	35 (80)	9	30
**Cameroon**	3 700	122	2010	351	247 (70)	104	83
**Central African Republic**	700	25	2009	295	215 (73)	80	109
**Chad**	2 300	79	2007	240	186 (78)	53	128
**Chile**	24 600	1749	2005	349	225 (64)	124	67
**China**	16 700	731	2012	289	178 (62)	112	43
**Colombia**	14 400	962	2011	370	248 (86)	122	48
**Congo**	6 800	323	2013	300	221 (74)	79	108
**Cook Islands**	16 700	486	2007	240	167 (70)	73	110
**Costa Rica**	16 900	1389	2014	388	225 (58)	163	4
**Côte d’Ivoire**	3 900	187	2014	502	266 (53)	236	−70
**Croatia**	24 700	1652	2010	599	286 (48)	313	−151
**Cuba**	12 300	2475	2012	506	282 (56)	225	−42
**Czechia**	35 500	2146	2012	802	264 (33)	538	−398
**Democratic People's Republic of Korea**	1 700	2060	2012	220	166 (75)	54	96
**Democratic Republic of the Congo**	800	32	2010	313	230 (74)	83	103
**Djibouti**	3 600	338	2007	199	150 (75)	49	105
**Dominica**	11 000	587	2007	284	202 (71)	82	136
**Dominican Republic**	17 000	580	2015	355	297 (84)	58	105
**Ecuador**	11 500	1040	2013	369	270 (73)	99	35
**Egypt**	12 700	594	2012	323	263 (81)	60	97
**El Salvador**	8 000	565	2009	360	253 (70)	107	82
**Eritrea**	1 600	51	2010	335	248 (74)	87	107
**Estonia**	31 700	1668	2012	405	156 (39)	249	−141
**Ethiopia**	2 200	73	2014	707	319 (45)	388	−209
**Fiji**	9 800	364	2015	296	215 (73)	81	114
**Gambia**	2 600	118	2001	164	126 (77)	38	92
**Georgia**	10 700	628	2007	247	206 (83)	41	139
**Ghana**	4 700	145	2010	302	219 (73)	83	104
**Grenada**	15 100	728	2007	282	197 (70)	85	130
**Guinea**	2 200	68	2012	238	194 (82)	44	116
**Guyana**	8 100	379	2010	280	216 (77)	64	116
**Haiti**	1 800	131	2012	197	182 (92)	15	153
**Honduras**	5 600	400	2009	365	227 (62)	138	25
**India**	7 200	267	2015	367	239 (65)	128	45
**Indonesia**	12 400	299	2011	275	222 (81)	53	127
**Iran (Islamic Republic of)**	20 100	1082	2014	886	342 (39)	544	−390
**Iraq**	16 700	667	2010	573	260 (45)	313	−143
**Jamaica**	9 200	476	2012	457	265 (58)	192	7
**Jordan**	9 200	798	2011	590	287 (49)	303	−138
**Kenya**	3 500	169	2016	416	310 (75)	106	30
**Kiribati**	2 000	184	2009	216	173 (80)	43	144
**Kyrgyzstan**	3 700	215	2009	316	206 (65)	110	56
**Latvia**	27 700	940	2012	304	127 (42)	177	−96
**Lebanon**	19 600	987	2014	284	232 (82)	52	108
**Lesotho**	3 300	276	2005	195	148 (76)	47	107
**Liberia**	1 300	98	2011	215	182 (85)	33	137
**Lithuania**	32 400	1718	2012	339	153 (45)	186	−77
**Madagascar**	1 600	44	2008	250	170 (68)	80	100
**Malawi**	1 200	93	2015	322	249 (77)	73	116
**Malaysia**	29 100	1040	2014	308	220 (71)	88	98
**Maldives**	19 200	1996	2011	535	243 (45)	292	−111
**Mali**	2 200	108	2012	285	220 (77)	65	127
**Malta**	41 900	3072	2008	607	245 (40)	362	−201
**Marshall Islands**	3 600	680	2007	214	142 (66)	72	80
**Mauritania**	4 500	148	2008	215	168 (78)	47	123
**Mexico**	19 900	1122	2011	706	294 (42)	412	−260
**Mongolia**	13 000	565	2009	256	216 (84)	41	126
**Montenegro**	17 800	888	2011	452	262 (58)	190	−26
**Morocco**	8 600	447	2012	344	252 (73)	92	78
**Mozambique**	1 300	79	2016	259	232 (90)	27	133
**Myanmar**	6 300	103	2010	315	249 (79)	67	137
**Namibia**	11 200	869	2016	382	262 (69)	120	72
**Nauru**	12 300	512	2010	230	177 (77)	53	132
**Nepal**	2 700	137	2011	300	242 (81)	58	116
**Nicaragua**	5 900	445	2011	271	212 (78)	59	125
**Nigeria**	5 900	217	2010	305	224 (73)	81	101
**Niue**	5 800	887	2006	213	141 (66)	72	75
**North Macedonia**	14 900	851	2008	390	218 (56)	172	−30
**Oman**	46 000	1442	2009	576	299 (52)	277	−94
**Pakistan**	5 400	129	2016	373	347 (93)	26	79
**Palau**	14 700	1429	2006	268	167 (62)	101	70
**Papua New Guinea**	3 700	109	2012	270	223 (83)	47	132
**Paraguay**	12 800	873	2009	306	224 (73)	82	92
**Peru**	13 500	656	2012	424	298 (70)	126	40
**Philippines**	8 400	329	2008	519	291 (56)	228	−45
**Poland**	29 600	1570	2017	441	177 (40)	264	−179
**Portugal**	30 500	2690	2011	905	256 (28)	649	−497
**Republic of Moldova**	6 700	514	2011	476	329 (69)	147	4
**Romania**	24 600	1079	2012	635	231 (36)	404	−283
**Russian Federation**	27 900	1836	2014	518	260 (50)	258	−118
**Rwanda**	2 100	125	2010	284	216 (76)	68	128
**Saint Kitts and Nevis**	28 200	1152	2007	290	204 (70)	86	140
**Saint Lucia**	14 400	698	2007	290	204 (70)	86	140
**Saint Vincent and the Grenadines**	11 500	917	2010	267	216 (81)	51	151
**Senegal**	3 500	107	2013	333	213 (64)	120	43
**Serbia**	15 100	1312	2010	472	237 (50)	235	−72
**Seychelles**	29 300	844	2010	294	210 (71)	84	114
**Slovakia**	33 100	2179	2012	983	291 (30)	692	−553
**Slovenia**	34 500	2698	2017	787	305 (39)	482	−359
**Solomon Islands**	2 200	108	2017	257	194 (75)	63	115
**Somalia**	1 064^c^	11	2006	82	73 (89)	9	66
**South Africa**	13 600	1148	2014	192	157 (82)	35	90
**Sri Lanka**	12 900	369	2013	318	230 (72)	88	62
**Sudan**	4 300	282	2014	300	175 (58)	125	34
**Suriname**	14 900	979	2014	285	220 (77)	65	113
**Sweden**	51 200	5219	2016	289	143 (49)	146	−61
**Syrian Arab Republic**	2 900	376	2008	964	312 (32)	652	−490
**Tajikistan**	3 200	185	2009	272	227 (83)	45	132
**Thailand**	17 900	600	2013	547	303 (55)	244	−67
**Timor-Leste**	6 000	102	2015	239	203 (85)	36	147
**Togo**	1 700	76	2012	295	234 (79)	61	109
**Tonga**	5 900	270	2007	229	164 (72)	65	123
**Trinidad and Tobago**	31 300	1816	2010	493	265 (54)	228	−41
**Tunisia**	11 900	785	2012	719	265 (54)	454	−283
**Tuvalu**	3 800	585	2010	177	150 (85)	27	139
**Uganda**	2 400	133	2012	363	247 (68)	116	71
**Ukraine**	8 800	584	2009	278	225 (81)	53	104
**United Republic of Tanzania**	3 200	137	2013	357	251 (70)	106	75
**Uruguay**	22 400	1792	2011	518	244 (47)	274	−106
**Vanuatu**	2 700	150	2006	177	147 (83)	30	131
**Venezuela (Bolivarian Republic of)**	12 500	923	2004	306	215 (70)	91	84
**Viet Nam**	6 900	390	2008	743	282 (38)	459	−289
**Yemen**	2 500	202	2009	247	201 (81)	46	131
**Zambia**	4 000	195	2013	286	217 (76)	69	92
**Zimbabwe**	2 300	115	2011	346	248 (72)	98	98

GDP: gross domestic product; Intl $: international dollars; WHO: World Health Organization.

^a^ Number of medicines on both WHO’s model list and the national list.

^b^ Number of medicines not on WHO’s model list.

^c^ Estimated GDP.

Note: WHO’s model list refers to WHO’s *Model list of essential medicines*.

**Fig. 1:**
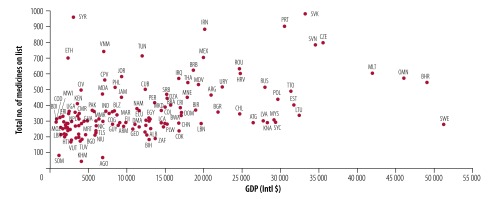
The number of essential medicines on national list of essential medicines in relation to countries’ gross domestic product, 2017

Fig. 1 shows the relationship between the number of essential medicines listed by each country and GDP. Most countries with a lower GDP had shorter national lists of essential medicines, but there were many exceptions. Sweden has a high GDP and relatively short list while Syrian Arab Republic has a low GDP and a relatively long list. Medicines in each country’s list can be found in a data repository.^17^^,^^18^

GDP: gross domestic product; Intl $: international dollars.

Note: We obtained the countries’ three letter codes from the International Organization for Standardization (ISO) 3166–1 Online Browsing Platform; AGO: Angola; ALB: Albania; ARG: Argentina; ARM: Armenia; ATG: Antigua and Barbuda; BDI: Burundi; BGD: Bangladesh; BGR: Bulgaria; BHR: Bahrain; BIH: Bosnia and Herzegovina; BLR: Belarus; BLZ: Belize; BRA: Brazil; BRB: Barbados; BWA: Botswana; CHL: Chile; CHN: China; CIV: Côte d'Ivoire; CMR: Cameroon; COD: Democratic Republic of Congo; COG: Congo; COK: Cook Islands; COL: Colombia; CPV: Cabo Verde; CRI: Costa Rica; CUB: Cuba; CZE: Czechia; DMA: Dominica; DZA: Algeria; ECU: Ecuador; EGY: Egypt; ERI: Eritrea; EST: Estonia; ETH: Ethiopia; FJI: Fiji; GEO: Georgia; GHA: Ghana; GUY: Guyana; HRV: Croatia; HTI: Haiti; IND: India; IRN: Islamic Republic of Iran; IRQ: Iraq; JAM: Jamaica; JOR: Jordan; KEN: Kenya; KHM: Cambodia; KNA: Saint Kitts and Nevis; LBN: Lebanon; LBR: Liberia; LCA: Saint Lucia; LTU: Lithuania; LVA: Latvia; MAR: Morocco; MDA: Republic of Moldova; MDV: Maldives; MEX: Mexico; MKD: North Macedonia; MLT: Malta; MMR: Myanmar; MNE: Montenegro; MOZ: Mozambique; MRT: Mauritania; MWI: Malawi; MYS: Malaysia; NAM: Namibia; NIC: Nicaragua; NIU: Niue; NPL: Nepal; OMN: Oman; PAK: Pakistan; PER: Peru; PHL: Philippines; PLW: Palau; POL: Poland; PRT: Portugal; ROU: Romania; SOM: Somalia; SVK: Slovakia; SVN: Slovenia; SWE: Sweden; SYC: Seychelles; SYR: Syrian Arab Republic; THA: Thailand; TTO: Trinidad and Tobago; TUN: Tunisia; TUV: Tuvalu; UGA: Uganda; URY: Uruguay; VNM: Viet Nam; VUT: Vanuatu; ZAF: South Africa. Not all country codes are shown to make this figure more readable; data for all countries are provided in Table 1.

### Comparison with WHO’s model list

Of the 414 eligible medicines on WHO’s model list, 73 (18%) medicines were listed by only 27 (20%) or fewer countries and 23 (6%) medicines were listed by 7 (5%) or fewer countries. Medicines recently added to WHO’s model list were generally listed by fewer countries than those medicines added earlier (available from a data repository).^19^ Only velpatasvir, a Hepatitis C treatment, which was added to the 2017 WHO model list , was not listed by any country. No country included all medicines on WHO’s model list; eight countries included over 300 WHO essential medicines on their list (Ethiopia, Iran [Islamic Republic of], Kenya, Pakistan, Republic of Moldova, Slovakia, Syrian Arab Republic and Thailand). Of these, Kenya, Pakistan and the Republic of Moldova listed WHO essential medicines without adding many (less than 150) other medicines. Portugal, Slovakia and Syrian Arab Republic added more than 600 medicines to their list that were not on WHO’s model list; while Angola, Bosnia and Herzegovina, Bulgaria, Cambodia and Somalia omitted more than 300 WHO essential medicines.

**Fig. 2:**
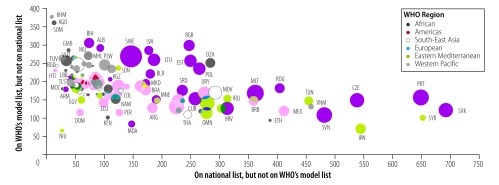
Health expenditure and dissimilarities between national lists of essential medicines and the 2017 WHO *Model list of essential medicines*

**Fig. 3:**
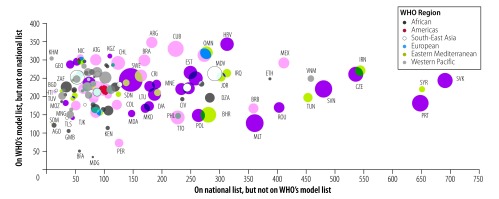
Health expenditure and similarities between national lists of essential medicines and the 2017 WHO *Model list of essential medicines*

Notes: The size of the circles represents the country’s health-care expenditure. We obtained the countries’ three letter codes from the International Organization for Standardization (ISO) 3166–1 Online Browsing Platform; AGO: Angola; ARG: Argentina; ATG: Antigua and Barbuda; BFA: Burkina Faso; BGD: Bangladesh; BGR: Bulgaria; BHR: Bahrain; BRA: Brazil; BRB: Barbados; BWA: Botswana; CHL: Chile; CIV: Côte d'Ivoire; COL: Colombia; CRI: Costa Rica; CUB: Cuba; CZE: Czechia; DZA: Algeria; EST: Estonia; ETH: Ethiopia; GEO: Georgia; GMB: Gambia; HRV: Croatia; HTI: Haiti; IRN: Islamic Republic of Iran; IRQ: Iraq; JOR: Jordan; KEN: Kenya; KGZ: Kyrgyzstan; KHM: Cambodia; LTU: Lithuania; LVA: Latvia; MDA: Republic of Moldova; MDG: Madagascar; MDV: Maldives; MEX: Mexico; MKD: North Macedonia; MLT: Malta; MNE: Montenegro; MNG: Mongolia; MOZ: Mozambique; NIC: Nicaragua; OMN: Oman; PER: Peru; PHL: Philippines; POL: Poland; PRT: Portugal; ROU: Romania; SDN: Sudan; SOM: Somalia; SVK: Slovakia; SVN: Slovenia; SWE: Sweden; SYR: Syrian Arab Republic; THA: Thailand; TJK: Tajikistan; TLS: Timor-Leste; TTO: Trinidad and Tobago; TUN: Tunisia; TUV: Tuvalu; VNM: Viet Nam; ZAF: South Africa. Not all country codes are shown to make this figure more readable; data for all countries are provided in Table 1.

The numbers of differences between each country’s list and WHO’s model list ranged from 85 to 533 (median: 252; IQR: 227 to 303) or, when equivalence to exemplars was disregarded, from 93 to 815 differences (median: 296; IQR: 265 to 381). There were differences across therapeutic areas and for both communicable and noncommunicable diseases (available from a data repository).^19^ Fig. 2 and Fig. 3 show the relationship between countries’ health-care expenditure and essential medicines. Countries with lower health-care expenditures appear to have omitted more medicines from their lists that are on WHO’s model list (e.g. Angola and Cambodia), and countries with higher health-care expenditures appear to have included more medicines on their lists that are not on WHO’s model list (e.g. Portugal and Slovakia), although exceptions exist (e.g. Sweden).

WHO: World Health Organization.

Notes: The size of the circles represents the country’s health-care expenditure. We obtained the countries’ three letter codes from the International Organization for Standardization (ISO) 3166–1 Online Browsing Platform; AGO: Angola; ALB: Albania; ARG: Argentina; ARM: Armenia; BGD: Bangladesh; BGR: Bulgaria; BIH: Bosnia and Herzegovina; BLR: Belarus; BRA: Brazil; BRB: Barbados; COL: Colombia; CUB: Cuba; CZE: Czechia; DOM: Dominican Republic; DZA: Algeria; ECU: Ecuador; EGY: Egypt; EST: Estonia; ETH: Ethiopia; GMB: Gambia; HRV: Croatia; HTI: Haiti; IRN: Islamic Republic of Iran; IRQ: Iraq; KEN: Kenya; KGZ: Kyrgyzstan; KHM: Cambodia; LBR: Liberia; LTU: Lithuania; LVA: Latvia; MDA: Republic of Moldova; MDV: Maldives; MEX: Mexico; MHL: Marshall Islands; MKD: North Macedonia; MLT: Malta; MNE: Montenegro; MOZ: Mozambique; NAM: Namibia; NIU: Niue; OMN: Oman; PAK: Pakistan; PER: Peru; PLW: Palau; POL: Poland; PRT: Portugal; ROU: Romania; SDN: Sudan; SOM: Somalia; SRB: Serbia; SVK: Slovakia; SVN: Slovenia; SWE: Sweden; SYR: Syrian Arab Republic; THA: Thailand; TLS: Timor-Leste; TUN: Tunisia; TUV: Tuvalu; URY: Uruguay; VNM: Viet Nam; VUT: Vanuatu. Not all country codes are shown to make this figure more readable; data for all countries are provided in Table 1.

**Fig. 4:**
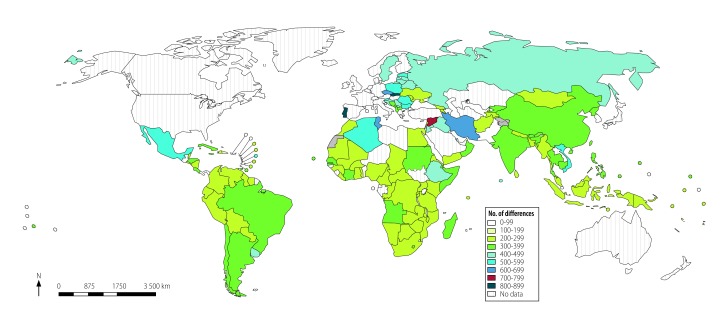
Differences between national lists of essential medicines and the 2017 WHO *Model list of essential medicines*

Notes: Differences mean that either a medicine is included on WHO’s model list, but not in that country’s list or a medicine is included on the country’s list, but not in WHO’s model list. No data indicates that no essential medicines list was found in the repository for that country.

**Fig. 5:**
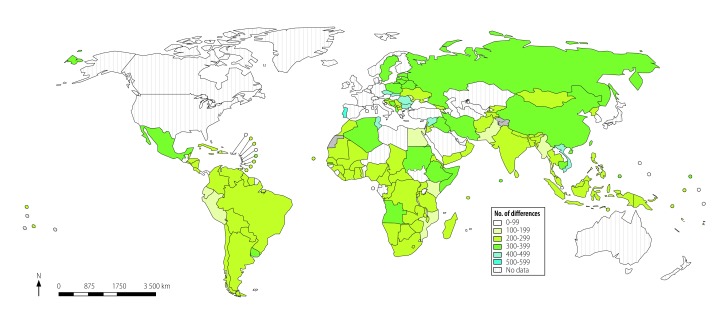
Differences between national lists of essential medicines and the WHO’s *Model list of essential medicines* when medicines in the same chemical subgroup are considered equivalent, 2017

Notes: Differences mean that either a medicine is included on WHO’s model list, but not in that country’s list or a medicine is included on the country’s list, but not in WHO’s model list. We used the Anatomical Therapeutic Chemical classification system to assess equivalence. No data indicates that no essential medicines list was found in the repository for that country.

The numbers of differences varied considerable within different WHO regions (Fig. 4). The differences between each country’s list and WHO’s model list across therapeutic areas were less when we consider equivalence based on Anatomical Therapeutic Chemical codes (Fig. 5). Algeria, Iran (Islamic Republic of), Mexico and Viet Nam are examples of countries listing large numbers of alternatives to the substances selected by WHO.

For the regression model, we included 136 countries. We excluded the country of Niue because of missing information. The multivariate linear regression indicated that the six included country characteristics explained one-third of the numbers of differences between each country’s list and WHO’s model list (adjusted *R^2^*:0.33); WHO region (more differences in the Americas) and health-care expenditure (more differences with higher expenditures) were significantly associated with the total number of differences (*P* = 0.023; available in a data repository).^19^ To determine if the main finding (that is, most of the variation in the number of differences was not explained by these country characteristics) depended on the definitions used in the pre-specified analysis, we conducted post-hoc sensitivity analyses. Excluding 17 countries with longer lists that may have been comprehensive formularies rather than essential medicines lists, although they were posted in the essential medicines lists repository, slightly increased the amount of variation in the number of differences explained by the country characteristics (*R^2^*: 0.37). Since long national lists will have many differences from WHO’s model list, we performed a sensitivity analysis accounting for list length using the Tanimoto coefficient and the *R^2^* decreased to 0.23 indicating that the main finding is not due to list length. Performing the same analyses using the 2015 WHO model list as the reference, rather than the 2017 version, showed no differences (median of the numbers of differences: 272; IQR: 244 to 367; *R^2^*: 0.33). 

### Between country comparisons

The similarity scores for countries, measuring the extent to which countries tend to list medicines commonly listed by other countries, ranged from −553 to 153 (median: 80; IQR: −45 to 115; Table 1).

Most of the medicines were listed by a relatively small proportion of the countries; 60% (1248/2068) of the medicines were listed by 10% (14) of the countries. Of these 1248 medicines, 250 (20%) were in the same main therapeutic area or the same anatomical subgroup as the closest related medicine on WHO’s model list; 349 (28%) medicines were in the same pharmacological subgroup as the most closely related medicine on WHO’s model list, 611 (49%) medicines were in the same chemical subgroup as the most closely related medicine on WHO’s model list, 30 (2%) medicines were on WHO’s model list, and 8 (1%) medicines could not be classified. The most commonly listed medicines are shown in Table 2. Amoxicillin was listed by all countries and diazepam, doxycycline, short-acting insulin, salbutamol, and metronidazole were each listed by 99% of countries.

**Table 2:** Most common medicines in countries’ lists of essential medicines, 2017

Medicine (synonym)	No. of countries list (%) *n* = 137
Acetazolamide	120 (88)
Acetylsalicylic acid	131 (96)
Acyclovir	129 (94)
Albendazole	112 (82)
Allopurinol	131 (96)
Amiodarone	118 (86)
Amitriptyline	127 (93)
Amoxicillin	137 (100)
Ampicillin	126 (92)
Atenolol	127 (93)
Atropine	127 (93)
Azithromycin	112 (82)
Beclometasone (Beclomethasone)	119 (87)
Benzylpenicillin (Penicillin G)	117 (85)
Betamethasone	126 (92)
Bupivacaine	116 (85)
Calcium	125 (91)
Carbamazepine	134 (98)
Carbidopa	116 (85)
Ceftriaxone	118 (86)
Chloramphenicol	117 (85)
Chlorpromazine	119 (87)
Ciprofloxacin	133 (97)
Clavulanic acid	116 (85)
Cyclophosphamide	114 (83)
Dexamethasone	131 (96)
Diazepam	135 (99)
Diclofenac	121 (88)
Digoxin	132 (96)
Dopamine	113 (82)
Doxycycline	135 (99)
Efavirenz	111 (81)
Epinephrine (Adrenaline)	128 (93)
Erythromycin	126 (92)
Ethambutol	126 (92)
Ethinylestradiol	117 (85)
Fentanyl	113 (82)
Ferrous fumarate	131 (96)
Fluconazole	125 (91)
Folic acid	132 (96)
Furosemide	133 (97)
Gentamicin	133 (97)
Glibenclamide (Glyburide)	122 (89)
Haloperidol	130 (95)
Heparin	125 (91)
Hydrochlorothiazide	130 (95)
Hydrocortisone	133 (97)
Ibuprofen	130 (95)
Insulin, long acting	115 (84)
Insulin, short acting	135 (99)
Isoniazid	127 (93)
Isosorbide dinitrate	119 (87)
Ketamine	113 (82)
Lamivudine	120 (88)
Levodopa	127 (93)
Levonorgestrel	112 (82)
Levothyroxine	130 (95)
Lidocaine (Lignocaine)	134 (98)
Magnesium	127 (93)
Mannitol	113 (82)
Medroxyprogesterone	119 (87)
Metformin	133 (97)
Methotrexate	126 (92)
Methyldopa	123 (90)
Metoclopramide	127 (93)
Metronidazole	136 (99)
Morphine	130 (95)
Naloxone	117 (85)
Neostigmine	119 (87)
Nifedipine	128 (93)
Nitroglycerin (Glyceryl trinitrate)	120 (88)
Nystatin	127 (93)
Omeprazole	127 (93)
Oxytocin (Pitocin)	123 (90)
Paracetamol (Acetaminophen)	133 (97)
Penicillin G Benzathine	119 (87)
Phenobarbital	131 (96)
Phenytoin	118 (86)
Pilocarpine	123 (90)
Potassium	128 (93)
Prednisolone	130 (95)
Propranolol	129 (94)
Pyrazinamide	126 (92)
Ranitidine	125 (91)
Rifampicin	129 (94)
Salbutamol	135 (99)
Spironolactone	131 (96)
Streptomycin	117 (85)
Sulfamethoxazole	130 (95)
Suxamethonium	111 (81)
Tamoxifen	116 (85)
Tetanus vaccine	115 (84)
Timolol	126 (92)
Trimethoprim	132 (96)
Valproic acid (Sodium valproate, Valproate, Valproate semisodium)	127 (93)
Verapamil	118 (86)
Vitamin B1 (Thiamine)	118 (86)
Vitamin B12 (Cobalamin)	125 (91)
Vitamin B6 (Pyridoxine)	125 (91)
Vitamin K (Menadione, Phytomenadione, Phytonadione)	122 (89)
Zidovudine (Retrovir)	118 (86)

Note: We classified medicines listed in more than 80% of national lists as common.

We examined medicines that were expected to be listed by only a small number of countries. There were six treatments for trypanosomiasis (pentamidine, suramin sodium, eflornithine, melarsoprol, nifurtimox, benznidazole) and four antileishmaniasis medicines (amphotericin B, miltefosine, paromomycin, sodium stibogluconate) on WHO’s model list. These medicines were listed by between eight and 96 countries (median: 12; IQR 9 to 24; more information available in a data repository).^19^

## Discussion

We found substantial differences in essential medicines lists. Most national lists of essential medicines had more than 200 differences compared with WHO’s model list. These differences were only partly explained by the countries’ characteristics we investigated. Most of the medicines were listed by a small number of countries. Decision-makers could choose to re-examine whether medicines listed by a small number of other countries should be removed from their national list.

Previous studies have compared many national lists of essential medicines, but for only one therapeutic area. For example, in one study on medications for neuropathic pain listed in the essential medicines lists of 112 countries, only four of 18 differences (22%) were related to country income.^20^ Gabapentinoids, that can be used to treat neuropathic pain, were more likely to be listed in high-income countries, although the efficacy of these medicines is questionsable.^21^^,^^22^ Other studies have compared lists of several countries for specific populations. For instance, comparing lists for paediatric populations have shown that the Indian and South African essential medicines lists may take better account of the needs of children compared with the Chinese list.^23^ The findings of these studies are consistent with our study, and also suggest that differences in the lists are not explained by countries’ characteristics, implying there may be opportunities to improve essential medicines lists.

Our study has limitations. We abstracted the medicines in each country’s list of essential medicines from the information posted on WHO’s website, a process that was liable to errors, as documents describing essential medicines lists had to be translated, standard medicine names were not consistently used, and judgements had to be made about what to include in ambiguous cases. In the future, stakeholders could validate and update the information in the data set used for this study and also provide information about how they are using the essential medicines list to the database of global essential medicines.^18^ Some of the lists included in this study may not be used by the respective countries. Furthermore, the country characteristics we included may not fully capture important features. We relied on the widely used Anatomical Therapeutic Chemical classification system, which like other classifications systems, assigns some medicines with multiple codes for different indications and does not include every medicine in use.

In 2004, WHO stated that the lack of access to essential medicines remains one of the most serious global public health problems and identified the “careful selection of essential medicine [as] the first step in ensuring access.”^24^ The importance of essential medicines lists will probably grow as countries move towards universal health coverage, as a part of achieving the sustainable development goals.^25^ Our findings suggest that greater care may be needed in selecting medicines that meet the priority health-care needs of populations. The reasons for the substantial differences from WHO’s model list and the differences between countries should be further studied. Governments could provide explanations for medicines they have decided to add to help other countries decide if they should also list them. Countries could also use the database of global essential medicines created for this study to flag medicines that are not listed by similar countries or in WHO’s model list.^18^ There may be gaps in the information available to countries about the medicines on WHO’s model list including the evidence supporting listing. Such additional information may help governments to decide if medicines on their lists should be removed or if other medicines should be added. WHO could also provide feedback to countries updating their lists on how their essential medicines lists compare with similar countries and highlight specific medicines for inclusion or removal, based on the decisions made by countries with similar health needs.

Many medicines are considered essential by only a small number of countries, and this difference is not likely explained by differences in health needs in those countries. Future work should determine whether specific changes should be made to particular essential medicines lists and explore the processes for creating and updating essential medicines lists. This may help identify opportunities to improve essential medicines lists and promote appropriate use of medicines in support of universal health coverage.
